# Punishing Wrongs from the Distant Past

**DOI:** 10.1007/s10982-019-09352-8

**Published:** 2019-03-21

**Authors:** Thomas Douglas

**Affiliations:** Oxford Uehiro Centre for Practical Ethics, University of Oxford, Suite 8, Littlegate House, 16-17 St Ebbes Street, Oxford, OX1 1PT, UK

## Abstract

On a Parfit-inspired account of culpability, as the psychological connections between a person’s younger self and older self weaken, the older self’s culpability for a wrong committed by the younger self diminishes. Suppose we accept this account and also accept a culpability-based upper limit on punishment severity. On this combination of views, we seem forced to conclude that perpetrators of distant past wrongs should either receive discounted punishments or be exempted from punishment entirely. This article develops a strategy for resisting this conclusion. I propose that, even if the perpetrators of distant past wrongs cannot permissibly be punished for the original wrongs, in typical cases they *can* permissibly be punished for their ongoing and iterated failures to rectify earlier wrongs. Having set out this proposal, I defend it against three objections, before exploring *how much* punishment it can justify.

Oskar Gröning worked as an SS administrator in the Auschwitz extermination camp during the Second World War. He was responsible for, among other things, guarding, sorting and logging the belongings of those who had been sent to the gas chambers or admitted to the camp.^1^ Gröning escaped punishment in the after-math of the war by concealing the nature of his wartime work, but in the 1980s he made his role at Auschwitz public. In 2015, following a change in German law that allowed for him to be prosecuted, he was tried as an accessory to the murder of at least 300,000 people. He was found guilty and sentenced to four years’ imprisonment,^2^ a decision that was upheld on appeal.^3^

Gröning’s sentence was not universally endorsed, even by those who testified against him.^4^ Commentators raised a number of questions. Was Gröning a victim of bad moral luck?^5^ Ought he to have been offered mercy given his frail condition, his (rather heavily qualified) expressions of remorse,^6^ and the fact that he had to an extent sought to undermine holocaust denial by speaking publicly about his experiences?^7^ And finally: did his wrongs simply lie too far in the past for a normal punishment to be warranted?

In this article, I address this last question, though in a more general form: should serious moral wrongs from the distant past be punished, and if so, should they be punished as severely as if they had been punished promptly? Or, as I will henceforth sometimes paraphrase this question, should distant past wrongs attract normal punishments?

My answer will be given in five stages. In §I, I present a previously noted, though as-yet-unexamined, argument for the view that distant past wrongs should not attract normal punishments – an argument for the view that perpetrators of distant past wrongs should be exempted from punishment or given less severe punishments than would have been warranted had they been punished promptly. In §II, I survey two possible replies to this argument, arguing that neither succeeds. In §III, I offer my own reply, which, I hold, at least partially undermines the case for punishment exemptions or discounts. In §IV, I respond to three objections to my reply. Finally, in §V, I consider *how far* my reply undermines the case for exemptions or discounts.

Before proceeding to these tasks, however, I need to make three preliminary remarks.

First, I am interested in the moral, not legal, justifiability of punishments for distant past wrongs. Moreover, I am interested not in whether such punishments can be morally justified under prevailing legal arrangements, but in whether they could be morally justified were the law to permit them, or were it possible, without cost, to reform the law in order to permit them. Many jurisdictions currently operate statutes of limitations that legally bar the punishment of at least some distant past crimes. If there is a moral obligation on states to comply with the law, and if those states cannot without cost reform the laws in question, they may have moral reasons not to punish distant past crimes. I set aside such reasons.

Second, I also set aside pragmatic limits on the degree to which punishments can be tailored to individual circumstances. Actual criminal justice systems must often, in part for pragmatic reasons, adopt sentencing policies that gloss over some differences between offenders. To give one example, they may need to adopt fixed minimum age limits for standing trial or receiving custodial sentences, even though it might be more desirable, absent practical constraints, to allow a case-by-case assessment of an individual’s degree of maturity. Such fine-grained assessment may simply be unreasonably costly, or too prone to biases or inter-assessor variations. I assume that there are no such constraints on punishment. On this assumption, it is no objection to reducing punishment in a particular case that we could not reasonably expect actual criminal justice systems to make the kind of assessment of individualized circumstances on which the reduction is based.

Third, I limit my focus to serious and culpable moral wrongs, for which the perpetrator has expressed no significant remorse, and which the perpetrator has made no significant attempt to rectify.^8^ I believe that these are the cases in which the moral case for punishing distant past wrongs is strongest, and I wish to present the view that distant past wrongs should attract normal punishments in its most plausible light.

## I A Challenge

Whether distant past wrongs should attract normal punishments may depend on the purposes that punishment serves. Let us assume, as a starting point, that the purpose of punishment is retributive: criminal punishment serves to mete out suffering that is proportionate to the offender’s culpability.^9^

At first sight, distant past wrongs may seem to pose no great problem for retributivism. If offenders are culpable for a wrong at the time they commit it, they plausibly remain so indefinitely, as long as they have not rectified their wrong. Culpability comes with no expiration date.

There is a complication, however. Suppose we accept a broadly Parfitian account of what matters in survival; suppose we accept that, as the psychological connections between a younger and older self weaken, the moral and prudential relationships between the younger and older self more closely approximate those between two different persons. Thus, for example, the younger self’s infliction of a harm on the older self becomes more and more similar, normatively speaking, to inflicting harm on another person, and the prevention of such a harm by a third party becomes more like a case of preventing harm to others, and less like a case of paternalism.

Parfit argues for this view – henceforth the ‘connectedness’ account of what matters in survival – chiefly by showing that, compared to competing accounts, it can better accommodate and rationalize our intuitive verdicts on a range of science fiction thought experiments.^10^ But the connectedness account also has some attractive implications in more everyday cases. For example, it is able to account for the widely held intuition that it would normally be less bad for a serious harm to befall one’s much older self than to befall one now. That is, it seems able to accommodate and rationalize our intuitive discounting of distant future harms.^11^ It also has possibly attractive implications for the morality of abortion; it is consistent with the view, which many find plausible, that abortion becomes wrong, and then more seriously wrong, as the fetus develops stronger psychological connections to the live-born person it will become.^12^

The connectedness account of what matters in survival is a view regarding how psychological connectedness affects morality and prudence in general. But, since it is meant to apply to *all* moral and prudential relationships, it entails a parallel, more specific view regarding how psychological connectedness affects *culpability* in particular. It entails that, as the psychological connections between a person’s younger and older selves weaken, the older self’s culpability for a wrong committed by the younger self diminishes. Call this the connectedness account of culpability.

Suppose we accept the connectedness account of culpability. And suppose we are now confronted with ‘the Octogenarian’, an 80-year-old who committed a serious and culpable wrong as a 20-year-old, but who is only weakly psychologically connected to his 20-year-old self. We may assume that the Octogenarian has few memories of, and shares few psychological traits with, his 20-year-old self, and that the 20-year-old had few expectations, hopes or desires regarding his 80-year-old self.

Is the Octogenarian fully culpable for his distant past wrong?

It is perhaps possible to maintain that he is, even if one thinks that psychological connectedness can affect culpability. One could, for instance, argue that there is a range over which the psychological connections between a younger and older self remain sufficiently strong that there is no reduction in culpability; there may be a threshold level of disconnectedness that must be reached before culpability begins to attenuate. Or one could hold that only loss of certain *kinds* of connectedness diminishes culpability. For example, it may be that culpability is reduced only when one distances oneself from one’s earlier wrong or rejects the motivations that produced it.

However, as the connectedness account of culpability is standardly understood, *any* significant reduction in psychological connectedness between an earlier and later self will be sufficient to cause *some* reduction in culpability for the earlier self’s wrong. This sort of view seems at one point to have been endorsed by Parfit himself. In *Reasons and Persons*, he speculated that ‘[w]hen some convict is now less closely connected to himself at the time of his crime, he deserves less punishment. If the connections are very weak, he may deserve none’.^13^ There is no hint here – or in the surrounding text – that culpability is diminished only once a certain threshold is crossed, or only when a certain kind of connection is lost.

On this interpretation of the connectedness account, which I will henceforth adopt, we must say either that the Octogenarian is not at all culpable for the wrong committed by the 20-year-old, or that only some of the 20-year-old’s culpability is inherited by the Octogenarian. If the former, any punishment would be disproportionate, so to conform to the requirements of retributivism, the Octogenarian would need to be exempted from punishment. If the latter, the maximal amount of punishment that qualifies as proportionate will be reduced, so, on retributivism, the Octogenarian ought to be given a reduced punishment (a ‘punishment discount’).^14^

This result can be generalized to any perpetrator of a wrong who is less psychologically connected to his self at the time of the crime than he would have been had he been punished promptly – something which is plausibly true of all actual perpetrators of distant past wrongs, as well as some perpetrators of more recent wrongs who have, since their wrongdoing, undergone a more sudden loss of connectedness, for example, due to radical memory loss or personality change.^15^ In any such case, there will, on the present elucidation of the connectedness account of culpability, have been a reduction in culpability, and we are assuming no pragmatic barriers to case-by-case tailoring of punishments to individual circumstances, including individual culpability.

The result can also be generalized to many theories of punishment that do not posit a retributive purpose for punishment. Many such theories nevertheless include a culpability-based upper limit on justified punishment. Thus, suppose the purpose of punishment is not to inflict proportionate suffering but is rather to prevent reoffending by the punished individual, deter offending by others, or communicate censure. Most would accept that there are constraints on how these purposes may permissibly be realized, and one constraint, plausibly, is that the punishment should not inflict more suffering than would be proportionate to the offender’s culpability. This is often referred to as the ‘negative retributivist constraint’, and I will adopt that terminology here. If culpability diminishes over time in the way I have been suggesting, the negative retributivist constraint will tighten over time – that is, the maximum amount of punishment that can be imposed without violating the constraint will be reduced. So again, on the connectedness account of culpability, either a punishment exemption or a punishment discount ought to be given.

## II Responding to the Challenge

Should we, then, give punishment discounts or exemptions to the perpetrators of distant past wrongs? In *Reasons and Persons*, Parfit took it to be plausible that we should, and offered a case to support this view:

Suppose that a man aged ninety, one of the few rightful holders of the Nobel Peace Prize, confesses that it was he who, at the age of twenty, injured a policeman in a drunken brawl. Though this was a serious crime, this man may not now deserve to be punished.^16^

However, it is not clear that this case supports the application of punishment discounts or exemptions *based on diminished psychological connectedness*. As Allen Buchanan has noted, it is possible to account for the plausibility of the view that a punishment discount or exemption should be applied in this case in other ways.^17^ We may be inclined to assume, for example, that through his good works the Nobel Laureate has, in effect, made some amends for, and thereby partly rectified, his earlier wrong. It may be that it is this partial rectification that justifies the discount.

Moreover, in other cases of distant past wrongdoing, it is less clear that punishment should be discounted. Recall the case of Oskar Gröning, with which we began. Gröning does not appear to have been actively involved in the killings at Auschwitz, and there is some evidence that he objected to some of what he witnessed there.^18^ But consider a variant of this case in which the former Nazi had been an active and enthusiastic participant in mass killings. Suppose also that this individual had done nothing to rectify his wrongs, showed no signs of remorse, and remained in excellent health at the time of sentencing. It is not intuitively clear to me that a punishment discount would be warranted in this case, even if the psychological connections between the younger Nazi and his much older self are weak.

Might it, then, be possible to resist the application of punishment discounts or exemptions for distant past crimes, even while endorsing the connectedness account of culpability? In this section, I consider two options for doing so.

The first option is to reject all theories of punishment that either posit a retributive purpose of punishment or incorporate a negative retributivist constraint. Rejecting such theories undercuts the positive case for punishment discounts or exemptions that I gave above.

This option is not appealing. For one thing, the thought that there is a culpability-based upper limit on justified punishment is an intuitively compelling one; punishing innocents and heavily punishing individuals who are only mildly culpable seem intuitively objectionable, and the existence of a negative retributivist constraint provides a credible explanation of their objectionability.^19^

Moreover, as it happens, the leading non-retributivist theories of punishment face other difficulties in justifying the punishment of distant past crimes even where they do not incorporate a negative retributivist constraint.

Consider what we may call ‘anti-recidivist’ penal theories, according to which the purpose of punishment is to prevent reoffending by the punished individual, whether through rehabilitation, incapacitation, or what is known as ‘specific’ deterrence – deterring the punished individual from future offending.^20^ On these theories, it is difficult to see why distant past crimes should typically be punished. It is unusual, in cases where a crime was committed long ago, that the perpetrator retains the disposition and ability to reoffend. Thus, the risk of recidivism will typically be too low to justify the costs of imposing punishment.^21^ Moreover, even if the offender does continue to pose a significant risk of recidivism, it is unlikely that we will possess sufficient evidence for this to justify punishment. Certainly, the mere fact that the individual has committed a wrong in the distant past will have little evidential value.^22^

Consider next the view that the purpose of punishment is general deterrence – deterring wrongdoing by others. Again, it seems unlikely that this goal would be advanced much by punishing wrongs from the distant past. Compare two different punishment schemes: in the first, all serious and culpable wrongs besides those committed in the distant past are, insofar as reasonably possible, punished; in the second, all serious and culpable wrongs, *including* those from the distant past, are punished, as far as reasonably possible. The second scheme can be expected to have a somewhat stronger deterrent effect than the first: potential wrongdoers will recognize, under that scheme, that they cannot avoid punishment by evading it until such time as their wrong lies in the distant past. However, there are two reasons to suppose that any additional deterrent effect associated with the second scheme will be slight. First, the number of additional wrongs punished under this scheme is likely to be very small, since there would presumably be very few offenders who would evade punishment until their wrongs lie in the distant past, and then succumb to it. Second, the additional punishments imposed under this scheme are, from the perspective of the time of the offence, punishments that lie far in the future. Given evidence that people tend to discount distant future harms,^23^ and that the deterrent effects of even prompt punishments are typically modest,^24^ such distant future punishments are unlikely to have a substantial deterrent effect on prospective wrongdoers. (The probable weak marginal deterrent effect of extending punishments to include distant past wrongs has, indeed, been invoked as basis for accepting statutes of limitations^25^).

Finally, consider communicative theories, according to which punishment serves to communicate censure. These accounts run into difficulties similar to those faced by retributivist accounts. On communicative accounts, censure must be communicated *to* an individual who is culpable for the crime. Thus, if we accept a connectedness account of culpability, perpetrators of distant past wrongs are either not an appropriate object of censure (since they are no longer at all culpable) or are no longer an appropriate object of such severe censure (because their culpability has been attenuated). A case for punishment discounts or exemptions would thus survive a move to a communicative theory, even if this theory were to eschew a negative retributivist constraint.

Let us turn, then, to a second option for resisting penal discounts or exemptions – an option alluded to, though not developed, by Parfit himself: we could argue that, even if the perpetrator of a distant past wrong is no longer culpable for that wrong, she is culpable as an accomplice to that wrong.^26^ This suggestion could be coupled with the recently defended view that accomplices are not, by virtue of being ‘merely’ accomplices, thereby less culpable than the principal agent of a wrong.^27^ The conjunction of these views would allow us to preserve the result that the older self is, or at least may be, as culpable as the younger self who committed the wrong, notwithstanding any reduction in connectedness.

The difficulty with this strategy is that, even if some kinds of complicity fully preserve culpability, in the sense that the accomplice is *ceteris paribus* just as culpable as the principal agent of the wrong, the specific kind of complicity involved in the cases of interest to us here does not. The most plausible explanations of why an older self is an accomplice to the wrong committed by her younger self are that (i) she benefits in some ways from that wrong, (ii) she to some degree shares the attitudes that motivated that wrongdoing, or (iii) some combination of (i) and (ii). However, even if we accept that one of these explanations succeeds, something many would deny,^28^ we would not normally regard the kind of complicity produced by such associations as fully culpability-preserving.^29^ Indeed, we would not normally regard them as conferring enough culpability to justify any punishment. Consider a case in which a parent commits a theft from which his child, as an adult, benefits. Suppose also that the child, as an adult, shares some of the attitudes that motivated the parent to commit the theft: perhaps, for example, she is just as disinclined to respect the law and the property rights of others. We would not, in this case, say that the child is as culpable as her parent. Indeed, we would not think that the child possesses any degree of culpability sufficient to justify punishment.

## III The Recursive Account

I propose a third strategy for resisting the application of punishment exemptions or discounts to perpetrators of distant past wrongs. My suggestion is that we might be permitted to punish such individuals not on the basis that they are culpable for the initial wrongdoing, nor on the basis that they are culpable accomplices to that wrongdoing, but on the basis that they have been guilty of ongoing wrongdoing in the intervening years.

Why think that the perpetrators of distant past crimes have been guilty of ongoing wrongdoing? My answer begins from the contention that, when one commits a serious and culpable wrong, one normally acquires a moral duty to rectify that wrong. I wish to remain neutral between different plausible views regarding what kinds of deeds can rectify a past wrong, but candidates would include mitigating, terminating or reversing the harm that one has wrongfully imposed; compensating for any harm that remains unmitigated; apologizing to one’s victim or her loved ones; expressing condemnation of or remorse for one’s past wrong; submitting to punishment for one’s wrong; and performing deeds that are in some sense comparable in moral significance to one’s wrong, but positive, rather than negative, in moral valence.^30^ People will reasonably disagree about whether, how, and to what degree different wrongs can be rectified. However, regardless what reasonable stance one takes on these matters, it is plausible that the perpetrators of the most serious and culpable moral wrongs can perform *some* deeds that would significantly rectify their wrongs, and that, where this is so, they often fall under a moral obligation to perform the said deeds, or a subset thereof, in order to (partially) rectify their wrong.

These thoughts suggest that if, as I have been assuming throughout, the perpetrator of a distant past wrong has never made any significant attempt at rectification, he is likely to have been guilty of a further wrong since his commission of the original wrong, namely, the wrong of failing to at least partially rectify that wrong.^31^

Now of course, if a connectedness account of what matters in survival holds, this obligation to rectify the initial wrong will dissolve over time as psychological connectedness weakens, just as culpability for the initial wrong weakens. Eventually, expecting someone to rectify a past wrong will be equivalent to expecting her to rectify someone else’s wrong. It may be, to return to our earlier example, that the Octogenarian no longer has any obligation to rectify the wrong he committed when he was 20. If so, then the Octogenarian commits no further wrong by failing to rectify his initial wrong. Still, it is plausible that, for some time beyond the age of 20, the Octogenarian *was* under an obligation to rectify his initial wrong.

To fix matters, let’s suppose that the Octogenarian’s obligation to rectify his initial wrong persisted for twenty years from the commission of that wrong. He remained under this obligation until the age of 40. Suppose further that during these 20 years, he did nothing to fulfil this obligation – nothing to rectify the wrong. He was thus guilty of a further moral wrong.

What do we say about this person beyond the age of 40? Do we have to say that he is no longer guilty of any wrongdoing, since he no longer has any obligation to rectify his initial wrong? Not obviously, since we can plausibly say that he still has a duty to rectify his secondary wrong; his wrong of failing to rectify the initial wrong. This is a wrong that he was still committing up until the age of 40, and it is plausible that he will, for some number of further years, remain under an obligation to rectify it. Again, to fix matters, let us suppose that this obligation will persist for a further 20 years and that, if he does nothing to rectify the wrong during these 20 years, he will be guilty of a further wrong – another wrong of failing to rectify an earlier wrong. This wrong is one that the Octogenarian was committing up to the age of 60 and will give rise to a new obligation to rectify that persists beyond that age.

We could keep iterating this reasoning and get the result that the Octogenarian has in fact been guilty of ongoing failures to rectify earlier wrongs. Initially, the failure was a failure to rectify the original wrong. Subsequently, the failures were failures to rectify earlier failures of rectification.

I will refer to the above explanation for why perpetrators of distant past crimes may remain culpable as the *recursive account*.^32^ I make two claims on behalf of this account. First, that it will often justify some punishment for perpetrators of distant past wrongs, even in cases where the individual is no longer at all culpable for the initial wrong. Second, that it will often diminish the punishment discount that should be applied to perpetrators of distant past wrongs who remain somewhat culpable for the initial wrong.

The case in favor of these claims is straightforward. If the recursive account is correct, then there is a plausible retributive justification for punishing the perpetrators of many distant past wrongs. Like our Octogenarian, these perpetrators remain culpable for at least some of their recent failures of rectification, so ought to be subjected to punishments that are proportionate to this culpability, over and above punishment they are due for any residual culpability for the initial wrong.

Similarly, the recursive account allows us to furnish credible antirecidivist, general deterrent and communicative justifications for punishment. In punishing the Octogenarian, for instance, we would arguably be forcing him to rectify his recent wrongs of non-rectification, thus putting an end to the ongoing wrongdoing. It is plausible that we would also significantly deter others from continuing to leave past wrongs unrectified, since the Octogenarian’s recent failures of rectification are, in effect, being promptly punished. Additionally, in punishing the Octogenarian, we would perhaps express appropriate censure for the wrong of leaving past wrongs unrectified. Moreover, the Octogenarian’s ongoing culpability for failures of rectification would help to loosen any negative retributivist limit that these theories impose.

Nevertheless, three significant objections could be raised to my claims, and it is to these that I now turn.

## IV Objections

The first of these objections holds that, even if failing to rectify a wrong is itself a wrong, it is not the sort of wrong for which punishment could be justified.

This objection might be supported by appealing to prevailing criminal justice practices. Our criminal justice systems frequently do treat failures of rectification as an aggravating factor in sentencing; punishment for a crime may be more severe if the offender has done little or nothing to rectify it. However, our criminal justice systems do not normally treat failures of rectification as independently punishable crimes, such that states could be justified in punishing failures of rectification even if, for one reason or another, they could not be justified in punishing the initial crime. To the extent that one thinks our criminal justice systems are somewhat responsive to moral reasons, one might think this provides some evidence for the view that failures of rectification are indeed not independently punishable wrongs.

On the other hand, however, none of the standard grounds for regarding a wrong as non-punishable clearly rules out the punishment of failures of rectification. For example, failures of rectification may be quite serious wrongs, so they could not universally be deemed unpunishable on the basis of their triviality. Similarly, wrongs of non-rectification do not always lie within the private sphere in a way that would set them beyond the proper sphere of outside influence. They are also not always such that their prohibition would be impossible or costly to enforce.

Moreover, the fact that current legal arrangements do not permit the punishment of failures of rectification does not, I think, have much, if any, evidential significance. After all, it may be that we do not currently punish failures of rectification because in almost all cases in which we might wish to do so, we can achieve the same result by simply treating them as aggravating factors in punishing the initial wrong. There are no commonly encountered circumstances in which we would *need* to punish failures of rectification as separate wrongs.

Let me turn, then, to the second and third objections; these raise a common set of issues, and I will deal with them together. The second objection holds that, if the recursive account justifies punishment, it justifies punishment of the wrong wrong. Intuitively, if the perpetrator of a distant past wrong ought to be punished, she ought to be punished for *that* wrong, not for subsequent failures of rectification.

The third objection maintains that my recursive account implausibly proliferates the number of wrongs that the perpetrator of a distant past wrong has perpetrated. In describing how the recursive account plays out in relation to the Octogenarian, I appealed to a series of three failures of rectification, each extending over a period of 20 years. But, of course, it would be possible, by shortening the length of each iteration, to describe many – perhaps infinitely many – alternative and overlapping series of wrongs, and it is not clear what basis we could have for preferring one series of iterated wrongs to the others. It might thus seem that the proponent of the recursive account is committed to holding that many perpetrators of distant past wrongs are guilty of committing an enormous number of wrongs.

These objections raise difficult questions about how to individuate wrongs that I cannot settle here. My response will instead be to simply note that there is *a* plausible way of delineating the wrongs in play in these cases that substantially de-fangs – even if it does not entirely undermine – these objections.

I have until now presented the initial wrong, the subsequent failures to rectify that wrong, and the further failures to rectify those failures of rectification, as distinct wrongs, but they may be closer to one another than my presentation to this point has suggested; these wrongs may all be constituents of a single composite wrong. Consider a case in which as a youth, a now-old man seriously assaulted another, and has subsequently done nothing to rectify either that wrongful assault or the intervening failures of rectification. Note, first, that, if we characterize them generically enough, the initial wrong and the subsequent failures of rectification can all be understood as wrongs of the same kind. We might, for example, characterize this kind of wrong as ‘acting so as to infringe, without rectification, a person’s rights’.^33^ Note, second, that, once characterized in this way, it seems plausible to regard these wrongs as parts of a single, temporally extended whole; the man initially acts so as to infringe, without rectification, the rights of his victim through committing an assault, and then continues to perpetrate that same wrong through his subsequent failures of rectification. Finally, note, third, that it is plausible that this overarching wrong is at least part of what we would have been punishing had we punished the man promptly, and also that this is the wrong we would now punish if we now follow the guidance of the recursive account. True, we would now be punishing a different *part* of the wrong than the part we would have punished had we punished the man promptly. But punishing different parts of a single wrong may seem less troubling than punishing entirely distinct wrongs.

In this way, subsuming the initial wrong and subsequent failures of rectification under a single, composite wrong diminishes the force of the objection that my recursive account recommends punishing the wrong wrong. It may also diminish the force of the concern that the account implausibly proliferates the number of wrongs that the wrongdoer commits, for if the manifold wrongs are all also parts of a single larger wrong, their great number seems less troubling. There are, after all, many familiar wrongs that can be decomposed into infinitely many sub-wrongs. Temporally extended wrongs of omission seem to be like this. Most of us plausibly commit the temporally extended wrong of failing to substantially alleviate the suffering of the poorest citizens of the world at low cost to ourselves, a wrong that consists, presumably, of many – perhaps infinitely many – less extended wrongs of the same kind.

There is also a second line of response to the second and third objections. Even if the recursive account *does* implausibly recommend punishing the wrong wrong and *does* implausibly proliferate the number of wrongs that the wrongdoer commits, it may nevertheless be correct.

One reason for this is that the recursive account may in fact *minimize* the overall counter-intuitiveness of the connectedness account of culpability. Given the connectedness account, which we are assuming, I think our choice may be between (1) rejecting the recursive account and accepting what many will regard as a counter-intuitively low level of punishment in cases like the modified version of the Gröning case that I discussed in section II, and (2) accepting the recursive account and thus endorsing an intuitively more acceptable level of punishment in such cases, although at the cost of accepting counter-intuitive implications regarding the number of wrongs committed and nature of the wrong for which punishment is being given. Depending on how we weigh these two kinds of counter-intuitiveness, the latter may be preferable.

## V How Much Punishment?

Suppose my claims regarding the recursive account hold good: the account can often justify some punishment, even in cases where the individual is not at all culpable for her distant past wrong, and it often diminishes the size of the punishment discount that should be given in cases where culpability for the initial wrong is reduced, but not to zero. A further question that arises is: *how far* does the account roll back the argument for penal discounts or exemptions? That is, how much (additional) punishment does the recursive account justify, when it justifies some?

I cannot develop a full answer to this question here, but I wish to end by mentioning four factors that will be relevant to answering it. In discussing these four factors, I will continue to refer to the initial wrong and the subsequent failures of rectification as distinct wrongs, even though I have just suggested that they can be subsumed under a single, overarching wrong. I take it that these can be separate wrongs while also being parts of a single, larger wrong. Any readers not convinced by this should interpret my references to these separate wrongs as references to parts of the larger wrong.

### A The Lesser Wrongness of Non-Rectification

The first factor that will influence the amount of punishment that can be justified, on the recursive account, is the degree to which culpability diminishes with each iteration of the cycle of wrongdoing and failure to rectify. This will depend in part on the relative seriousness of (i) committing a wrong, and (ii) failing to rectify it.

Suppose that failing to rectify a wrong is typically a less serious wrong, other things being equal, than was the wrong that remains unrectified (I henceforth refer to this view as the lesser wrongness of non-rectification). In that case, we might expect that a person’s culpability will normally diminish with every iteration of the cycle of wrongdoing and failure to rectify.

The lesser wrongness of rectification yields intuitively plausible verdicts on at least some cases. Suppose that the day before yesterday I stole an innocent person’s phone.^34^ Then, yesterday, an opportunity arose to rectify this wrong by returning the phone, issuing an apology, and compensating for the inconvenience. I did not take this opportunity. On which day did the most serious wrong occur, yesterday or the day before? The day before yesterday, I would think.

Moreover, several principled arguments can be offered for the lesser wrongness of non-rectification. Let me mention just one, which appeals to the thought that most wrongs cannot be fully rectified. Let us say that a wrong is fully rectified if not committing the wrong is morally equivalent, in the overall seriousness of the wrong(s) committed, to committing the wrong and then rectifying it. Most wrongs cannot be fully rectified in this way. This suggests that committing a wrong will typically be a more serious wrong than failing to rectify it simply because it has a greater moral opportunity cost. The best alternative to committing a wrong (that is, not committing it) is better than the best alternative to not rectifying a wrong (that is, partially rectifying it). Thus, committing the wrong involves a greater moral loss relative to the best available alternative.

### B Repetition/Temporal Extension

The lesser wrongness of non-rectification holds that, other things being equal, failing to rectify a wrong is (typically) less wrong than the initial wrong. But other things are not equal in my recursive story. Whereas the initial wrong, in cases of distant past wrongdoing, is typically a single, short-lived wrong, the failures of rectification invoked by my recursive account are most plausibly understood as either recurrent or temporally extended wrongs. It is not that an individual commits a wrong and then commits a one-off short-lived further wrong of failing to rectify that wrong. Rather, she commits many individual wrongs (which we might think of occurring when an opportunity to rectify the wrong arises and is not taken), or a single but ongoing wrong that extends over many years.

The repeated and/or temporally extended nature of these failures of rectification plausibly increases their overall seriousness. Other things being equal, it makes them more serious than one-off, non-extended wrongs, and this will in turn tend to make the perpetrator’s culpability greater.

Precisely how much influence this factor exerts on the amount of punishment that may be imposed will depend on complex questions regarding how we individuate failures of rectification, and how we aggregate seriousness of wrongdoing – and overall culpability – across these failures (or across time, if the failures are temporally extended).

On the question of individuation: Should we say that the wrongdoer commits a further separate wrong on each day, or each hour, or each minute that he could (partially) rectify the wrong and fails to do so? Or should we think of the wrong of non-rectification as a continuous, temporally extended wrong? Does wrongdoing occur only at times where the possibility of rectifying the past wrong comes to the agent’s mind, or might reasonably be expected to do so? Or is the wrongdoer continuously guilty of this wrong?

On the question of aggregation: Are two equivalent wrongs of non-rectification collectively precisely twice as serious as each alone? (Alternatively, assuming that the wrong is instead a temporally extended one, is twice the length of temporally extended wrongdoing precisely twice as serious?) Or is the increase in seriousness of wrongdoing over time (or with repeat occurrences) sub- or supra-linear?^35^ Further, does *culpability* aggregate in the same way as seriousness of wrongdoing?

I cannot resolve these questions here. Instead, I will limit myself to making four hopefully uncontroversial observations.

First, the repeated and/or temporally extended nature of the wrongs of non-rectification does increase their (collective) seriousness relative to a situation in which there was only a single non-extended failure to rectify.^36^ It thus tends to offset the considerations mentioned in the previous sub-section, which suggested that a one-off failure to rectify a wrong is typically less seriously wrong than the initial wrong.

Second, because the repeated and/or temporally extended nature of the wrongs of non-rectification tends to increase their seriousness, it tends also to increase culpability; other things being equal, the perpetrator of a more serious wrong or set of wrongs is more culpable than the perpetrator of a less serious wrong or set of wrongs.

But – and here is my third observation – the repeated and/or temporally extended nature of failures of rectification may also tend to increase culpability in another way. In cases of one-off time-limited wrongdoing, an agent’s culpability is often diminished by the fact that she had little time to reflect before deciding whether to perform the wrongful action. The lack of time may provide a partial excuse. It is much less likely that this excuse would apply in cases of repeated or temporally extended wrongdoing.^37^

Finally, fourth, in at least some cases, it seems plausible that, even over a quite limited period of time, a person’s aggregate culpability for failure(s) to rectify a wrong is comparable to her culpability for the initial wrong. Consider a case in which *A* seriously assaults *B* and is then, over the subsequent five years, repeatedly presented with opportunities to substantially rectify this wrong by compensating, and issuing an apology to, these victims. Suppose further that *A* is financially well off, so that offering the compensation would cost her little. In this sort of case, it might seem that *A*’s failures of rectification are, in aggregate, at least as serious as her initial wrong, and that her culpability for the failures of rectification is at least as great as that for the initial wrong, leaving aside any diminishment of initial culpability due to loss of psychological connectedness.

### C The Rate of Psychological Disconnection

I have identified two competing factors bearing on the amount of punishment that my recursive account can justify: the lesser wrongness of non-rectification will tend to diminish the amount of punishment that the account can justify; the repeated and perhaps temporally extended nature of the failures of rectification invoked by the account militates in the opposite direction. A third factor affects the balance between these two factors: the rate at which culpability and the obligation to rectify previous wrongs attenuate over time due to loss of psychological connectedness. This will affect the balance between the two aforementioned factors in two ways.

First, it will affect the number of iterations of wrongdoing and failure to rectify that it is necessary to invoke to justify the claim that the perpetrator remains culpable. The more rapidly obligations to rectify attenuate, the more iterations we will need to invoke to get the result that, for instance, our Octogenarian remains culpable. If, due to the lesser wrongness of non-rectification, each iteration results in some loss of culpability, a greater number of iterations will tend to lead to a greater loss in culpability over time, and thus a reduction in the amount of punishment that the recursive account can justify.

Second, the rate at which culpability diminishes over time will affect how far back we can look in punishing the perpetrator of the distant past wrong. For instance, if culpability diminishes very quickly, it might be that our Octogenarian can be punished only for failures of rectification that he has committed over the preceding 5 years. If it diminishes slowly, it may be that he can be punished for failures of rectification over the previous 40 years, say.

In order to determine the amount of (additional) punishment that can be justified by my recursive account we need, then, to balance (i) the lesser wrongness of non-rectification against (ii) the repeated and/or temporally extended nature of the failures of rectification invoked by the recursive account, in the light of (iii) the rate at which culpability and obligations to rectify attenuate over time due to loss of psychological connectedness.

### D Degree of Rectification

In many actual cases where the punishment of distant past wrongs is under consideration, a further factor will need to be considered: to what extent has the offender in fact rectified his earlier wrongs? I have, in this article, assumed throughout that the older self has expressed no remorse for the wrong of his younger self and has done nothing (else) to rectify it. But most real cases are not like this. Oskar Gröning, as I noted, offered some qualified expressions of remorse, and used his experiences to undermine holocaust denial. Both could be regarded as partially rectifying his earlier wrong, though I find it implausible that they do so to a great extent. Still, if there is partial rectification here, this will somewhat diminish the amount of punishment that can be justified on my account.

## Footnotes

1 Matthias Geyer, ‘An SS Officer Remembers: The Bookkeeper from Auschwitz’, *Spiegel Online* (09/05/2005), <http://web.archive.org/web/20070302085046/http://www.spiegel.de/international/spiegel/0,1518,355188,00.html>.

2 *See* ‘Oskar Gröning’, *Trial International* (08/05/2016), <https://trialinternational.org/latest-post/oskar-groning/>. There have been two trials of Auschwitz personnel since Gröning’s conviction. In 2016, Reinhold Hanning, who had been an SS guard at Auschwitz, was convicted of aiding and abetting the murder of at least 170,000 people, though he passed away before an appeal could be heard, so never began the five-year prison term to which he was sentenced. A third Auschwitz trial, of former SS medic Hubert Zafke, was terminated due to Zafke’s illness. On Hanning, *see* ‘Reinhold Hanning’, *Trial International* (09/08/2016), <https://trialinternational.org/latest-post/reinhold-hanning/> and ‘Reinhold Hanning: Convicted Nazi Guard Dies Before Going to Prison’, *BBC News* (01/06/2017), <http://www.bbc.co.uk/news/world-europe-40122610>. On Zafke, *see* ‘Hubert Zafke’, *Trial International* (29/11/2016), <https://trialinternational.org/latest-post/hubert-zafke/> and ‘German court stops trial of paramedic who worked at Auschwitz’, *Reuters* (12/09/2017), <https://www.reuters.com/article/us-germany-nazi-trial/german-court-stops-trial-of-paramedic-who-worked-at-auschwitz-idUSKCN1BN1E6>.

3 ‘Bundesgerichtshof: Halfstrafe Gegen Oskar Gröning Ist Rechtskräftig’, *Zeit Online* (28/11/2016), <http://www.zeit.de/gesellschaft/zeitgeschehen/2016-11/bundesgerichtshof-oskar-groening-urteil-ns-massenmord>.

4 Melanie Hall, ‘‘Holocaust Survivor Criticises German Court for Decision to Jail ‘Bookkeeper of Auschwitz’,’’ *The Telegraph* (15/07/2015), <http://www.telegraph.co.uk/news/worldnews/europe/germany/11742517/Holocaust-suvivor-criticises-German-court-for-decision-to-jail-bookkeeper-of-Auschwitz.html> and Kashmira Gander, ‘Auschwitz Survivor Criticised for Saying SS Guard Oskar Groening Should Not Be Prosecuted’, *The Independent* (28/04/2015), <http://www.independent.co.uk/news/world/europe/oskar-groening-trial-auschwitz-survivor-eva-mozes-kor-criticised-by-co-plaintiffs-for-saying-ex-ss-10208797.html>.

5 *See*, Roger Crisp, ‘The Luck of Oskar Groening’, *Practical Ethics Blog* (23/04/2015), <http://blog.practicalethics.ox.ac.uk/2015/04/the-luck-of-oskar-groening/>.

6 Gröning has admitted to feeling guilt for his role at Auschwitz and to having requested forgiveness from God but he has also denied that he was an active perpetrator in the killings, expressed reluctance to accept the label of ‘accomplice’, and claimed that his participation was involuntary. *See* Geyer, ‘An SS Officer Remembers: The Bookkeeper from Auschwitz’, *supra* note 1.

7 Geyer, ‘An SS Officer Remembers’, *supra* note 1.

8 I believe that it is debatable whether Gröning’s case falls within the scope of this limitation and will take no stance on this.

9 I use the unqualified term ‘retributivist’ to refer to what could more precisely be called ‘positive retributivist’ views. I return to negative retributivism below.

10 Derek Parfit, *Reasons and Persons* (Oxford: Oxford University Press, 1984), ch. 13.

11 Parfit notes that his view can rationalize such discounting, though he does not present it as an advantage of the view. *See* his *Reasons and Persons*, §§103-5, *supra* note 10.

12 Again, Parfit notes this possible implication of his view, but does not clearly present it as an attractive implication. *See* his *Reasons and Persons*, §108, *supra* note 10.

13 Parfit, *Reasons and Persons*, p. 326, *supra* note 10.

14 I assume here that there is no respect in which the perpetrator of the distant past wrong has become *more* culpable over time, an assumption that I will later question.

15 In the following discussion, though, I focus only on cases in which psychological connections are weakened gradually across an extended period of time. The arguments I make would, I think, play out quite differently in cases of sudden psychological change.

16 Parfit, *Reasons and Persons*, p. 326, *supra* note 10.

17 Allen Buchanan, ‘Advance Directives and the Personal Identity Problem’, *Philosophy & Public Affairs* 17(4) (1988): pp. 277–302, at p. 291.

18 Gröning claims that he requested transfer away from the camp on a number of occasions, though it is not clear whether this was motivated by discomfort with the means via which some killings were carried out or discomfort with the fact of the killings. *See* Geyer, ‘An SS Officer Remembers: The Bookkeeper from Auschwitz’, *supra* note 1.

19 It might be thought that it is sufficient, for the purposes of accommodating these intuitions, to accept a *defeasible* variant of the negative retributivist constraint—a constraint that allows for disproportionate punishments in exceptional cases. However, even a defeasible variant of the constraint will be sufficient to justify punishment discounts or exemptions for *most* distant past wrongs. Thus, shifting to such a constraint does little to diminish the impact of the connectedness account of culpability.

20 I include under this banner views according to which punishment is justified as a form of self-defense.

21 Allen Buchanan makes the same point in relation to Parfit’s Nobel Prizewinner case. *See* Buchanan, ‘Advance Directives and the Personal Identity Problem’, *supra* note 17.

22 Even where distant past wrongdoing has no evidential value, it could play a role in justifying anti-recidivist punishment. For example, this history may make the offender morally liable to anti-recidivist measures by removing or loosening a deontological constraint that would otherwise render them permissible.

23 For a review, *see* e.g., Tobias Kalenscher and Cyriel M.A. Pennartz, ‘Is a Bird in the Hand Worth Two in the Future? The Neuroeconomics of Intertemporal Decision-Making’, *Progress in Neurobiology* 84(3) (2008): pp. 284–315.

24 Two recent reviews of the evidence support this claim. *See* Daniel S. Nagin, ‘Deterrence: A Review of the Evidence by a Criminologist for Economists’, *Annual Review of Economics* 5 (2013): pp. 83– 105; and Aaron Chalfin and Justin McCrary, ‘Criminal Deterrence: A Review of the Literature’, *Journal of Economic Literature* 55(1) (2017): pp. 5–48.

25 *See*, for this argument, Yair Listokin, ‘Efficient Time Bars: A New Rationale for the Existence of Statutes of Limitations in Criminal Law’, *The Journal of Legal Studies* 31(1) (2002): pp. 99–118.

26 *See* Parfit, *Reasons and Persons*, p. 326, *supra* note 10, and, for discussion, Rebecca Dresser, ‘Personal Identity and Punishment’, *Boston University Law Review* 70 (1990): pp. 395–446, at pp. 425–27.

27 Chiara Lepora and Robert E. Goodin, *On Complicity and Compromise* (Oxford: Oxford University Press, 2013), p. 34, esp. note 9; Katrien Devolder, ‘U.S. Complicity and Japan’s Wartime Medical Atrocities: Time for a Response’, *The American Journal of Bioethics* 15(6) (2015): pp. 40–9, at p. 44, note 4; Victor Tadros, ‘Permissibility in a World of Wrongdoing’, *Philosophy & Public Affairs* 44(2) (2016): pp. 101–32.

28 That these kinds of association confer complicity could be denied on the grounds that complicity requires causal contribution. *See*, for this view, John Gardner, ‘Complicity and Causality’, *Criminal Law and Philosophy* 1(2) (2007): pp. 127–141; Lepora and Goodin, *On Complicity and Compromise*, *supra* note 27; and Björn Petersson, ‘Co-Responsibility and Causal Involvement’, *Philosophia* 41(3) (2013): pp. 847–866.

29 Dresser, ‘Personal Identity and Punishment,’ pp. 425-427, *supra* note 26, argues for a similar but stronger claim. She suggests that one can be culpably complicit in another’s wrong only if one ‘chose to participate’ (p. 427) in that wrong. A future self can, she claims, make no such choice in relation to a past self’s wrong.

30 For illuminating discussions of various candidate forms of rectification, *see* e.g., Stephen P. Garvey, ‘Punishment as Atonement’, *UCLA Law Review* 46 (1998): pp. 1801-1858; Philip Pettit, ‘Republican Theory and Criminal Punishment’, *Utilitas* 9(1) (1997): pp. 59–79; Todd N. Karhu, ‘Non-Compensable Harms’, *Analysis*, online (2018).

31 It might be claimed that, not only is the failure of rectification a further wrong, it also aggravates the initial wrong, for example, by increasing the amount of harm that it causes to the victims and their loved ones. *See*, for discussion of a similar claim, Nick Smith, *Justice Through Apologies: Remorse, Reform, and Punishment* (New York: Cambridge University Press, 2014), esp. at pp. 186–187. My subsequent argument does not require me to take any view on whether failures of rectification aggravate the initial wrong.

32 For somewhat similar explanations of why present people or nations bear obligations in relation to historical injustices, *see* Bernard R. Boxill, ‘A Lockean Argument for Black Reparations’, *The Journal of Ethics* 7(1) (2003): pp. 63–91; George Sher, ‘Transgenerational Compensation’, *Philosophy & Public Affairs* 33(2) (2005): pp. 181–200; Daniel Butt, ‘Nations, Overlapping Generations and Historic Injustice’, *American Philosophical Quarterly* 43(4) (2006): pp. 357–367; and Andrew I. Cohen, ‘Compensation for Historic Injustices: Completing the Boxill and Sher Argument’, *Philosophy & Public Affairs* 37(1) (2009): pp. 81–102. Note that these authors are partly, and in the case of Sher and Cohen primarily, concerned not with how a perpetrator’s culpability is transmitted forward in time, but rather with how a victim’s claim to compensation is transmitted forward in time—and, in the case of interest to these authors, across generations. The transmission of the claim to compensation could also be an issue in the cases of interest to me here *if* one thinks (i) that rectification for a crime must take the form of compensating its original victim *and either* (ii) the victim(s) of the original crime have since died, *or* (iii) there has been a weakening in psychological connectedness between the victim’s younger and older self. I am inclined to reject (i), and thus to regard the transmission of claims to compensation as a non-issue in the cases of interest to me here. However, I am, unsurprisingly, sympathetic to the view that, if it is an issue, it can be resolved in the way suggested by these authors.

33 I do not claim that all of the wrongness of the initial wrong can be satisfactorily captured by this generic specification; perhaps there are also other kinds of wrongs committed initially. For example, perhaps part of the initial wrong consists specifically in *initiating* a rights infringement.

34 I choose a theft as the initial wrong because this is arguably the type of wrong that is most fully rectifiable, and in relation to which it is thus perhaps most plausible to think that failures of rectification would be as seriously wrong as the initial wrong.

35 One reason to think that the increase might be sub-linear, in at least some cases, is that, intuitively, repeated or temporally extended minor wrongdoing cannot give rise to a serious overall wrong even when repeated many times or extended over a long period. One reason to think that the increase might be supra-linear in some cases is that earlier wrongs might be thought to generate a special obligation not to commit further similar wrongs in the future. For a similar suggestion, *see* Christian Barry and Gerhard Øverland, ‘The Implications of Failing to Assist’, *Social Theory and Practice* 40(4) (2014): pp. 570–90.

36 This could be the case where the opportunity to rectify arises only on a single occasion.

37 Similar considerations have been offered in support of the view that first-time offenses should attract a punishment discount. *See* e.g., Andrew von Hirsch, *Past or Future Crimes: Deservedness and Dangerousness in the Sentencing of Criminals* (Manchester: Manchester University Press, 1986), esp. at pp. 83–85; Andrew von Hirsch, ‘Criminal Record Rides Again’, *Criminal Justice Ethics* 10(2) (1991): pp. 2–5.

